# Appetite

**Published:** 1885-03

**Authors:** 


### APPETITE.

Appetite is entirely distinct from hunger, which may be painful in its extent; it is nature's intimation that something is needed to supply the place of that which has been used or taken away.

Every motion of the body, every thought of the mind, wears away and uses up some particles of the tissues, and unless other particles are supplied in their place, the parts would be worn out and lose their strength, and the system would cease to work,

Appetite is in the nature of instinct, a faithful monitor, which will not cease its admonitions until the want is supplied, until something has been eaten.

The best and most healthy appetite is that which inclines us to eat in moderation when the regular time for eating comes.

If the appetite is voracious day after day it is the appetite of disease, and instead of gratifying it freely, we should curb it. The easiest way to correct such a voracious appetite as is sometimes connected with dyspepsia, is to take half as much as usual, eat it slowly, and in half an hour you feel that you are not hungry at all, and had eaten enough.

Persistence in this method will in a reasonable time break up the voraciousness, unless some other cause than dyspepsia is at the root of it.

